# Heterostructured TiO_2_/SiO_2_/γ-Fe_2_O_3_/rGO Coating with Highly Efficient Visible-Light-Induced Self-Cleaning Properties for Metallic Artifacts

**DOI:** 10.1021/acsami.0c06792

**Published:** 2020-06-03

**Authors:** Maryam Mokhtarifar, Reyhaneh Kaveh, Mojtaba Bagherzadeh, Andrea Lucotti, MariaPia Pedeferri, Maria Vittoria Diamanti

**Affiliations:** † Department of Chemistry, Materials and Chemical Engineering “Giulio Natta”, Politecnico di Milano, Milan 20133, Italy; ‡ Department of Chemistry, Sharif University of Technology, Tehran 11365-11155, Iran

**Keywords:** self-cleaning, TiO_2_, γ-Fe_2_O_3_, SiO_2_, reduced graphene oxide, superhydrophilic photocatalyst, transparent coating

## Abstract

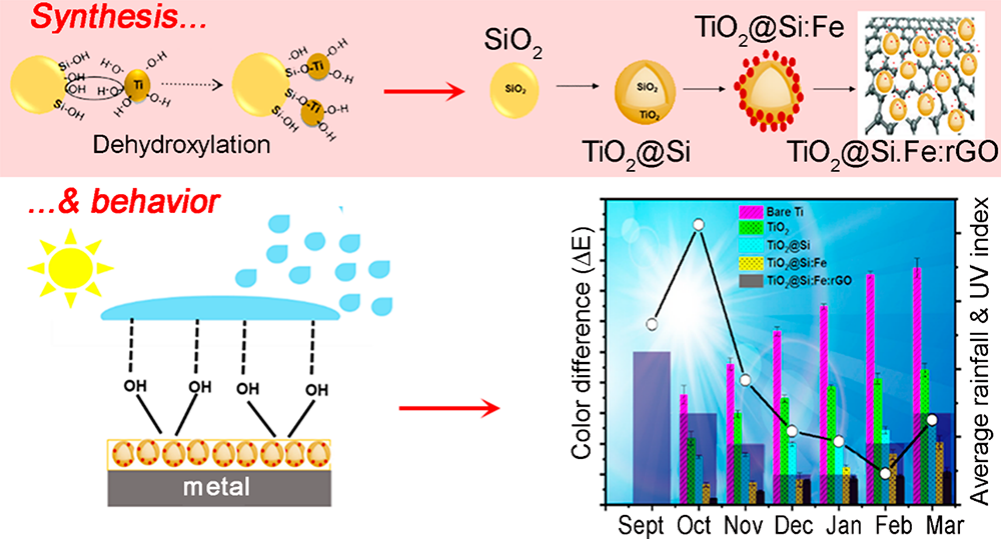

A novel nanohybrid composite of TiO_2_, SiO_2_, γ-Fe_2_O_3_, and reduced graphene oxide (TiO_2_@Si:Fe:rGO) is fabricated by the sol–gel method. The properties of the coated film were examined by structural and self-cleaning analyses using simulated discoloration/soiling and roofing tests. The fabricated transparent TiO_2_@Si:Fe:rGO composite showed excellent photoactivity and wettability, behaving well in self-cleaning applications. The addition of SiO_2_ improved the crystalline structure and surface hydroxylation of TiO_2_ nanoparticles. γ-Fe_2_O_3_ decreased the recombination rate of e^–^/h^+^ pairs, and significantly improved photocatalytic activity under visible light. Moreover, rGO sheets as excellent electron acceptors and transporters also reduced recombination, as well as affected wettability, achieving superhydrophilicity under irradiation. The coated substrate showed excellent resistance to simulated acid rain and significantly preserved the substrate from soiling in roofing tests.

## 1 Introduction

Preservation of current and future artifacts that have left a valuable visual art legacy is of great importance in today’s world. However, atmospheric deterioration continues to threaten tangible architectural heritages, especially those buildings and monuments made out of metals that are highly prone to various types of outdoor declines, including degradation and discoloration.^1−5^

To overcome this challenge, many studies have been dedicated to the elimination of pollutants by using photoactive metal oxides such as WO_3_, ZnO, and TiO_2_.^6−9^ Among them, TiO_2_ is one of the most considerable materials in the built environment due to its high photocatalytic efficiency and good integration with building materials. Indeed, its physicochemical properties, such as high chemical stability, easy availability, and the possibility to be easily added as coating or in mass to cementitious materials or to be deposited in the form of a thin film and reaching the superhydrophilic state, make it a great candidate for the preservation of metallic artifacts. The effect is that of reducing maintenance time and cost and protecting them from soiling by means of its self-cleaning features.^9−13^ Besides, some of the general aspects of suitable coatings to preserve metallic historic and modern buildings exposed to harsh environmental situations are proper adhesion, chemical stability, colorlessness, and transparency to maintain the aesthetic characteristics of the original material.^14^

To furtherly increase the photocatalytic efficiency, it is essential to engineer TiO_2_ hydrophilic surfaces to maximize the removal of contaminants and counteract soiling and discoloration. However, the wide band gap of the semiconductor for both anatase and rutile forms of TiO_2_ limits both its hydrophilic conversion and photocatalytic activity and, consequently, its self-cleaning ability to happen only at 5% of the solar spectrum, i.e., the UV region of sunlight.^15^

Within this context, heterojunction structures that combine other narrow band gap metal oxides with TiO_2_ can be used to achieve better light-harvesting and charge transport properties. On the other hand, the presence of the metal oxides in TiO_2_ composites could promote the electron transfer reactions between the photocatalyst and pollutant molecules. Moreover, materials with high specific surface area such as carbon-based materials can be utilized as an excellent supporting material to amplify photoactivity by increasing available surface sites.^16,17^

Trying to face this challenge, the present work aims at the synthesis and characterization of a nanocomposite coating including silicon dioxide (SiO_2_), γ-iron oxide (γ-Fe_2_O_3_), and reduced graphene oxide (rGO)) on titanium substrates. The synergistic effects of the components above with TiO_2_ are investigated in terms of resulting film morphological, structural and functional characteristics with special reference to self-cleaning practical applications.

## 2 Materials and Methods

### 2.1 Preparation of Sols and Coatings

Iron chloride tetrahydrate (FeCl_2_. 4H_2_O), propylene oxide, tetraethyl orthosilicate (TEOS), tetrabutyl titanate (TBT), sulfuric acid (H_2_SO_4_), graphene oxide solution (GO), sodium borohydride (NaBH_4_), ethanol (C_2_H_5_OH), and methylene blue (MB) were purchased from Sigma–Aldrich and used as received. To prepare the γ-Fe_2_O_3_ nanoparticles, FeCl_2_.·H_2_O (5 mmol) was dissolved in ethanol (0.3 M) and then added to propylene oxide (50 mmol), sonicated for 15 min, and followed by stirring for 6 h. Then, the resulting solution was heated at around 100 °C to obtain a brown powder.

To synthesize rGO, NaBH_4_ (6 mmol) was dissolved in diluted water (0.3 M) and then the solution was sonicated for 10 min. The aqueous solution was added dropwise to 5 mL of homogeneous GO aqueous dispersion (2 mg/mL), while it was being stirred at 80 °C for 1 h. The suspension was centrifuged and washed several times to obtain a black powder of rGO.

A homogeneous TiO_2_@Si:Fe:rGO sol–gel was synthesized by adding TEOS (40 mmol) to 65% HNO_3_ and stirring the solution for 1 h (the molar ratio of TEOS and H^+^ was kept 2.4) followed by centrifuging and drying at room temperature for 10 h. The obtained powder was dissolved in 10 mL of ethanol under sonification for 1 h, and then propylene oxide (400 mmol) and TBT (40 mmol) were added to the solution to form solution A that was then aged for 72 h. Solution A was mixed with 0.032 g of as prepared γ-Fe_2_O_3_ nanoparticles, and the mixture was sonicated for 1 h to obtain solution B. Finally, 0.02 g of the obtained rGO powder and solution B were mixed via stirring for 2 h and the obtained solution was named as TiO_2_@Si:Fe:rGO (for some comparison, a solution including just TiO_2_ was prepared as well). The concentration of TiO_2_ and SiO_2_ components followed the work of Kwon et al.^18^

Supporting Information Figure S1 shows a schematic representation of the TiO_2_@Si:Fe:rGO synthesis. In this figure, it can be observed that the SiO_2_ and TiO_2_ particles are bound by -Si–O–Ti- bonds. The strength of the chemical bond is stronger than that of van der Waals forces and other physical forces, so the coating of nano-TiO_2_ on a SiO_2_ surface is stable.^19^ The TiO_2_@Si:Fe nanoparticles then lie on the sheets of rGO.^20,21^

The final sol produced, TiO_2_@Si:Fe:rGO and the intermediate sols TiO_2_, TiO_2_@Si, and TiO_2_@Si:Fe were then applied to metallic substrates, consisting of commercial purity titanium specimens, grade 2 ASTM, with dimensions 50 mm × 50 mm and thickness 0.5 mm. All specimens were coated in the TiO_2_@Si:Fe:rGO sol with the dip-coating method. Although spray coating could be more applicable in the field of cultural heritage preservation as well as building materials functionalization, dip coating was selected in this work in order to have a better control on the process, resulting an uniform thickness and morphology.^22−24^ These properties could be adjusted with changing some parameters such as immersion/withdrawal speeds, angle, and number of dip coatings.^25−29^ However, because the solution is watery, it could be easily applied with spray-coating technique as well.

The number of dips was always set to be two, and the immersion rate was kept at 200 mm/min while the withdrawal rate was modulated at 100 mm/min. Careful consideration was taken into account for setting the bottom edge of the substrate to the solution surface, as well as dipping angles (90°). To have coated substrates with the same geometric area, a selected area of 1.7 × 1.9 cm^2^ was exposed and the rest of the sample was covered with Kapton tape. The process was done with a drying phase in ambient conditions for 24 h between each dipping followed by calcination at 600 °C for 2 h.

### 2.2 Characterization

All of the materials were characterized using field emission scanning electron microscopy (FE-SEM), X-ray fluorescence (XRF), X-ray diffraction (XRD), Raman spectroscopy, Fourier transform infrared (FTIR) spectroscopy, atomic force microscopy (AFM), UV–vis–near-IR diffuse reflectance spectroscopy, and optical microscopy (OM) to understand their structure, optical properties, photocatalytic performances, and the stabilities of the coated films.

The morphologies of the powder samples (TiO_2_@Si and TiO_2_@Si:Fe:rGO) were evaluated by FESEM (TESCANMIRA ΙΙ). For samples preparation, the synthesized nanoparticles were dispersed in ethanol and then a drop of mixed solution was withdrawn and dried on an aluminum plate. XRD was used to investigate the crystalline structures in the coated samples; diffraction patterns were recorded on a Philips PW1830 powder diffractometer operating at 40 kV voltage and 40 mA filament current. Spectra were acquired at the scanning rate of 2.5 deg/min with Cu Kα_1_ radiation in the 2θ range 20–60°. The weight fractions of anatase and rutile were calculated according to eq 1,^30^ where *I*_R_ and *I*_A_ are the intensity of the strongest reflections for rutile and anatase in (110) and (101) phases, individually.
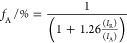
1For the composition analysis XRF was employed (ARL PERFOMIR’X). The optical properties were investigated by UV–vis–near-IR diffuse reflectance spectra, recorded in the 220–2600 nm range with a Shimadzu UV 3600 Plus spectrophotometer equipped with an ISR-603 integrating sphere, and BaSO_4_ was used as the reference material. The band gap was calculated on the basis of the reflectance UV–vis spectra after Kubelka–Munk conversion using the Tauc plot method.^31^ The FT-IR spectra of the prepared samples were recorded on a Bruker Tensor 27 spectrometer using a KBr pellet for sample preparation at room temperature. The Raman spectra were measured with a LABRAM HR800 equipped with a Peltier cooled CCD detector, and λ = 514 nm excitation was done by an argon ion laser (Stabilite 2017, Spectra-Physics). The laser radiation was filtered by an interference filter and focused on the sample by an Olympus BX41 microscope. A 50× Olympus objective with a 0.7 numerical aperture was utilized. The Rayleigh radiation was rejected using Notch filters for the λ = 514 nm laser line. Surface roughness of the coated samples was studied by atomic force microscopy (AFM) using a NT-MDT AFM Solver Pro apparatus operating in contact mode on an area of 60 μm × 60 μm. The coated samples were deposited on titanium specimens with an initial mirror polish surface finish to allow the investigation of the roughness associated with the composite deposition. Optical microscopy (OM) was used to assess possible cracking of the film before and after photodegradation test, in order to give a first evaluation of film structural stability (Leica INM 200). In addition, adhesion was evaluated by comparing OM images before and after making a scratched line at the coated samples using a diamond tip.

To evaluate self-cleaning properties, the wettability of the coated thin films was examined from the contact angle (CA) of a water droplet. These measurements were done on five predefined positions by a CCD camera connected to the computer via a PCI card. For each sample, the average contact angle was measured both under dark and after illumination for 30 min. Measurements were repeated after discoloration tests to check whether the coatings maintained their self-cleaning attitude also after possible discoloration.

### 2.3 Photocatalytic Degradation Performance

The photocatalytic activity of the coated thin films was characterized in methylene blue degradation under UV and visible light irradiation by spectrophotometry (UV–vis spectrophotometer, Thermo Scientific Spectronic 200E) at the wavelength 668 nm where MB has its maximum absorbance.

In a typical experiment, the MB solution was prepared by adding dye to deionized water (10^–5^ M), and then the coated specimens (1.7 × 1.9 cm^2^) were put in the MB solution (40 mL). Before photodegradation, the specimens were kept in the dark for 50 min to reach adsorption–desorption equilibrium. The samples were then illuminated for 6 h under UV LED (500 mA, 3.8 V) and visible LED (700 mA, 3 V) with 3 cm LED–sample distance. Eventually, the photodegradation of MB was calculated as a function of the relative MB concentration versus time of reaction, *C*/*C*_o_, where *C*_o_ is the initial concentration of MB solution and *C* is the concentration at sampling time, as derived from absorption values. Pseudo-first-order kinetics was hypothesized to control the rate of MB photodegradation, as observed in related scientific literature,^32,33^ so the slope of the curve of ln(*C*/*C*_o_) versus time of reaction was taken as MB photodegradation rate constant *k*.

### 2.4 Simulated Artificial Soiling

The typical composition of soiling present on urban surfaces is mostly including four soiling agents that were selected in this experimental work to mimic natural soiling: black carbon, mineral dust, humic acids (organics), and inorganic salts. The four soiling agents were mixed in different ratios in an aqueous mixture and applied to the samples’ surfaces. Dip deposition of the soiling agents with 45° dipping angle was chosen for ease of control and because soot deposition is typically waterborne.^34^ These individual components were selected to simulate soiling of metallic roofings exposed in four different climates representative of different cites with modern and contemporary titanium-based artifacts that have varying polluted atmospheres^35,36^ (Figure S2 and Table 1).

**Table 1:** Volume Fractions of Soiling Mixtures Used to Mimic the Soiling of Roofing Materials

		soiling components (%)			
site	artifact	carbon black	humic acid	dust	salt
Cleveland, OH, USA	Peter B. Lewis building	8	0	61	31
Glasgow, United Kingdom	Glasgow Science Centre	6	15	52	27
Normandy, France	Juno Beach Centre	12	36	28	24
Patras, Greece	Patras museum	5	8	63	24

To study the self-cleaning ability of the coated titanium specimens, they were put under solar light for 1 h to be photoactived and then dipped in the soiling components immediately. The soiled plates were left to dry; then the change in their color due to soiling was measured by a portable spectrophotometer Konica Minolta 2500d working in reflectance mode and compared with their initial color.

Roofing test measurements were taken from September to March 2019, in Milan, Italy. The samples were exposed outdoors by fixing them on a rack that was placed on the roof of a building, 45° tilt angle, facing South. The colors of specimens were monitored for 6 months at the beginning of each month.

Color changes before and after the tests were evaluated in terms of color difference, according to the L*a*b* color system: Δ*E* = √((ΔL*)^2^ + (Δa*)^2^ + (Δb*)^2^); the larger the color difference Δ*E*, the more significant was the discoloration. Here, symbols ΔL*, Δa*, and Δb* refer to the differences in L*, a*, and b* values, respectively, of the samples before and after tests.

### 2.5 Simulated Acid Rain Test

Metals freshly exposed to atmosphere as building roofs and walls are immune to pitting or crevice corrosion; nevertheless, these metals may undergo color changing after prolonged exposure. In the case of titanium, discoloration is a result of the formation of oxide films on the surface, which show interference colors by interacting with light.^4,37,38^ It is proved that discoloration is accelerated in the presence of titanium carbide (TiC) on the titanium surface, which is introduced in the sheet during the manufacturing process.^39^ Even though color changing does not adversely affect the structural function of the metal components, it sometimes spoils the building appearance.^40,41^ Furthermore, it is proved that the rate of discoloration changed depending on the building location and material factors, even though different sheets were adjacent to each other on the same building.^40^

To evaluate discoloration resistance of the samples, bare and coated titanium pieces were immersed in solutions of prescribed pH values simulating acid rain, obtained by adding H_2_SO_4_ and NaOH to distilled water. Other environmental factors such as air-borne salts and ultraviolet rays are not investigated here because they were found to have a marginal effect on metals discoloration.^37^ The solution pH for the immersion tests was controlled in a range of 3–7 by changing the ratio of sulfuric acid and sodium hydroxide, and the specimens were immersed in the solution kept at 65 °C for 7 days to accelerate the procedure.

## 3 Results and Discussion

### 3.1 Structure of the Coated Films

**Figure 1:**
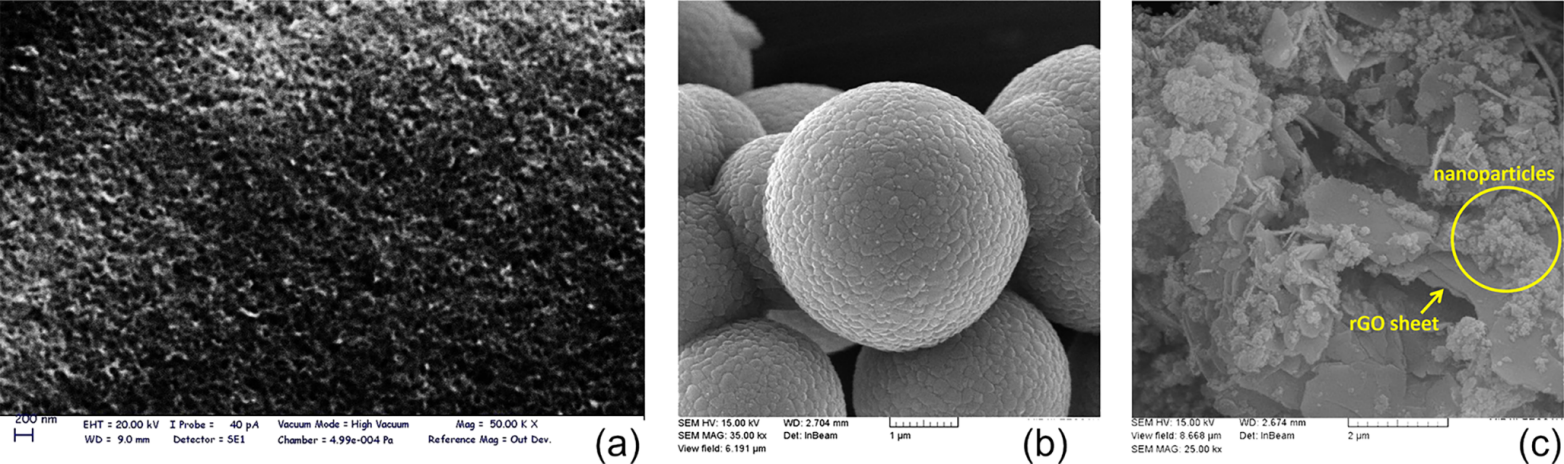
SEM images of (a) structure of TiO_2_@Si:Fe:rGO thin film, (b, c) distribution of the nanoparticles in TiO_2_@Si and TiO_2_@Si:Fe:rGO composites, respectively.

FESEM morphology images of synthesized composites are reported in Figure 1. On the basis of Figure 1a, TiO_2_@Si:Fe:rGO thin film shows apparently a uniform coating structure though more detail on particles distribution could be figured out from powder SEM morphology. Hence, images of the TiO_2_@Si and TiO_2_@Si:Fe:rGO composite powders are presented in Figure 1a,b, respectively.

Figure 1b reveals that TiO_2_ nanoparticles have smaller diameter and are dispersed on SiO_2_ particle surfaces uniformly. In panel c, it could be observed that particles of TiO_2_, SiO_2_ and γ-Fe_2_O_3_ are well distributed on the surface of the rGO nanosheets and well served as substrate for the homogeneous distribution of the nanoparticles.

XRF analysis results are presented in Table 2. This table shows the chemical compositions in the sample of TiO_2_@Si:Fe:rGO.

**Table 2:** XRF Analysis Results of the TiO_2_@Si:Fe:rGO Sample

sample	TiO_2_	SiO_2_	Fe_2_O_3_	Na_2_O	Al_2_O_3_	K_2_O	MnO	P_2_O_5_	LOI
analysis (%)	38.79	34.85	0.915	0.1	0.09	0.07	0.01	0.009	25.127

According to the obtained results, the atomic ratios Ti/Si and Ti/Fe were calculated as 0.83 and 44, respectively.

**Figure 2:**
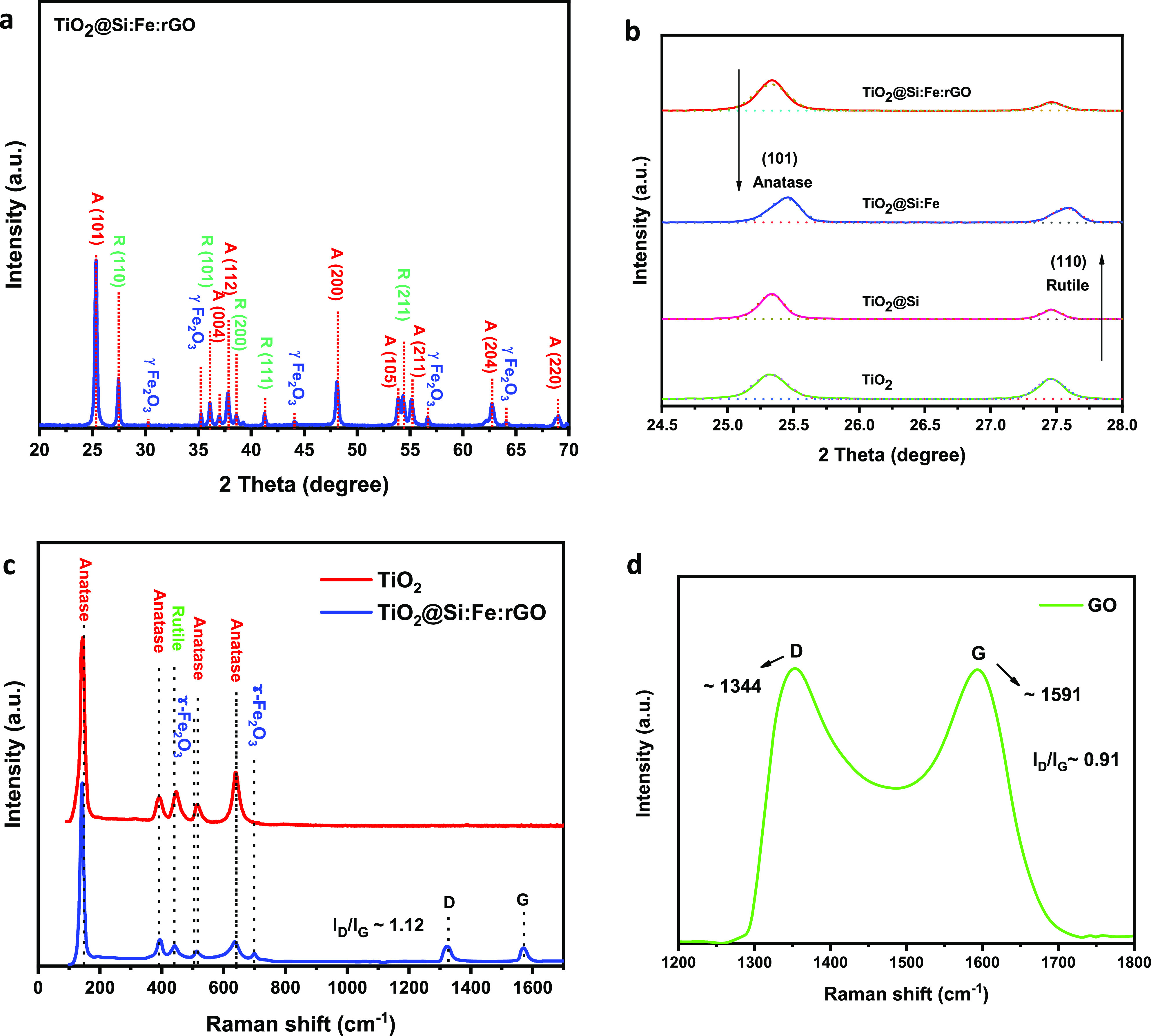
XRD patterns of (a) TiO_2_@Si:Fe:rGO coated film and (b) anatase (101) and rutile (110) peaks of the samples after heat treatment at 600 °C. Raman spectra of (c) TiO_2_ and TiO_2_@Si:Fe:rGO and (d) GO samples in the wavenumber range from 100 to 1800 cm^–1^.

XRD patterns and crystalline structures of the coated samples calcined at 600 °C are presented in Figure 2a,b and Table 3, respectively. It can be observed that the addition of SiO_2_ to TiO_2_ enhanced the thermal stability of anatase TiO_2_ crystallites, resulting in the retardation of the anatase-to-rutile phase transition. Indeed, the presence of SiO_2_ can prevent the nucleation of rutile.^42^ No peak for silica was observed; hence, SiO_2_ is amorphous. Crystallite sizes obtained by Scherrer equation were about 31 and 27 nm for the samples of TiO_2_ and TiO_2_@Si calcined at 600 °C, respectively. From literature it is known that for this transition to occur, anatase particles need to grow to a sufficient size, anatase being thermodynamically more stable for small nanoparticles. However, the contribution of bulk energy to total energy increases along with particle size. The transition to rutile takes place in a reconstructive process to decrease the total energy. In this way, the presence of SiO_2_ prevented phase transition by inhibiting the growth of the primary particles. The presence of SiO_2_ around TiO_2_ could efficiently delay the growth of nanoparticles likely due to the formation of the Ti–O–Si bond and because of the presence of amorphous SiO_2_ in contact with TiO_2_, which would hinder the growth of particles of TiO_2_.^43−45^

**Table 3:** Anatase Percentage in Crystalline TiO_2_ (Calculated by Spurr Equation) and Anatase Particle Size (Calculated by Scherrer Equation)

			particle size (nm)	
sample	anatase (%)	rutile (%)	anatase	rutile
TiO_2_	54	46	31	40
TiO_2_@Si	64	36	28	42
TiO_2_@Si:Fe	59	41	24	36
TiO_2_@Si:Fe:rGO	71	29	23	33

In the TiO_2_@Si:Fe composite, since the ionic radius of Fe^3+^ (0.64 Å) is similar to the ionic radius of Ti^4+^ (0.68 Å), the replacement of iron in the matrix of titania is not only possible but also favorable.^46^ Thus, structural deformations and the presence of defects in the TiO_2_ crystals might have formed by the substitution of Ti^4+^ by Fe^3+^, which marginally reduces the crystallinity of TiO_2_.^47^ In the presence of Fe_2_O_3_ in the TiO_2_@Si:Fe composite, anatase TiO_2_ peaks decrease in intensity while peaks for rutile phase start appearing. This observation indicates that Fe(III) ions have substantial influence on the anatase titania phase in binary TiO_2_–Fe_2_O_3_ compounds. The incorporation of Fe_2_O_3_ in the titania matrix might have facilitated the anatase–rutile phase transformation, indicating the presence of Fe^3+^ ions as substitutes in titania crystals.^46−51^

Also, the XRD pattern of this sample shows diffraction peaks corresponding to the (220), (311), (400), (511), and (440) planes of γ-Fe_2_O_3_ (Figure 2b)^52^ but no separate peak for rGO in the composite, possibly due to the low amount and low intensity of rGO. Moreover, the characteristic peak of rGO at 24.5° may be screened by the main peak of anatase TiO_2_ at 25.3°.^50^

Interestingly, TiO_2_ phase transformation from anatase to rutile was retarded, as the anatase/rutile ratio increased by adding rGO in the sol; hence, the presence of carbon retarded the anatase-to-rutile transformation, as already reported in literature related to activated carbon.^50^ This may be attributed to the high surface area of rGO, as well as to a reduction of acidity and promotion of the dehydration of Ti^4+^ complex, by forming edge shared bonds, thus producing anatase phase by adding rGO in the nanocomposite.^51,53^ Indeed, a variation in the number of OH ligands in the Ti complex produced from Ti^4+^ hydrolysis was observed to change acidity.^52,54,55^

Crystallographic structures of the prepared samples were also investigated by Raman spectroscopy and exhibited in Figure 2c,d. Scatterings at 145, 393, and 638 cm^–1^ are characteristic of anatase, while the one at 445 cm^–1^ is related to rutile.^42^ Given a smaller crystallite size and consequent relaxation of surface atoms, which are lacking adjacent atoms, peaks are slightly shifted in TiO_2_@Si:Fe:rGO.^48^ For this sample both characteristic bands of D and G peaks of GO, located at 1323 and 1570 cm^–1^, have shifted to lower frequencies in comparison with pure GO. This issue can be good evidence that GO was successfully reduced. Also, the intensity of *I*_D_/*I*_G_ ratio peaks of TiO_2_@Si:Fe:rGO compared to pure GO was enhanced. It can be related to the reduced average size of sp^2^ domains during the reduction process of GO in TiO_2_@Si:Fe:rGO nanocomposite. Moreover, peaks at 513 and 700 cm^–1^ were attributed to the presence of γ-Fe_2_O_3_ in this nanocomposite.^56^

**Figure 3:**
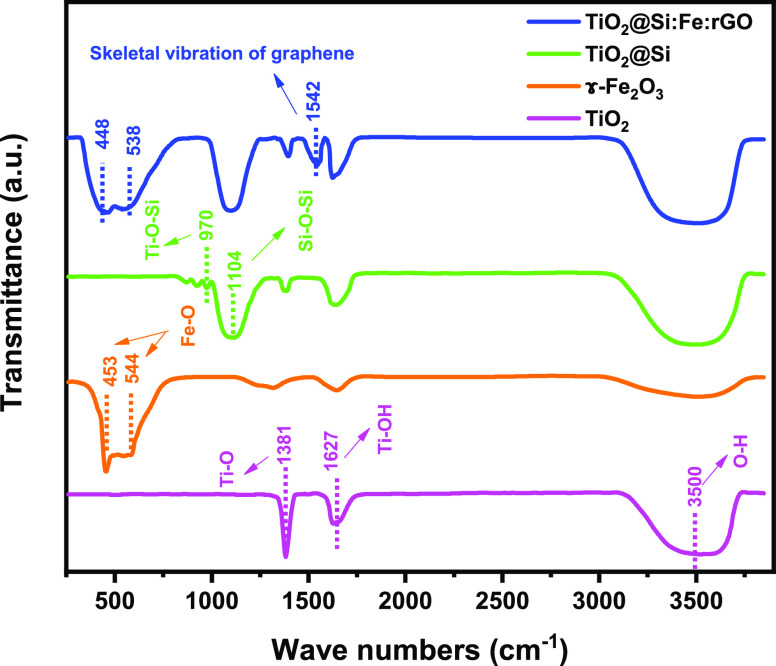
FT-IR spectra of TiO_2_, TiO_2_@Si, γ-Fe_2_O_3,_ and TiO_2_@Si:Fe:rGO samples in the wavenumber range from 300 to 4000 cm^–1^.

The FT-IR spectra of the TiO_2_, TiO_2_@Si, γ-Fe_2_O_3_, and TiO_2_@Si:Fe:rGO samples is shown in the wavenumber range from 300 to 4000 cm^–1^ (Figure 3). TiO_2_ shows three bands including a broad absorption band at around 3500 cm^–1^, related to the stretching vibration of hydroxyl groups O–H bound to the surface of TiO_2_, an absorption band at 1627 cm^–1^, due to bending of Ti–OH, and an absorption band at 1381 cm^–1^ for Ti–O modes. In TiO_2_@Si, at ∼970 cm^–1^ Ti–O–Si bonds appear, which are responsible for delayed anatase-to-rutile transformation (see XRD results).^57,58^ Moreover, the absorption band at 1104 cm^–1^ is attributed to the asymmetric Si–O–Si stretching vibration. γ-Fe_2_O_3_ shows strong bands at 453 and 544 cm^–1^ (Fe–O groups); these peaks were shifted to 448 and 538 cm^–1^ in TiO_2_@Si:Fe:rGO probably due to surface augmentation.^59^ Eventually, in TiO_2_@Si:Fe:rGO, the absorption peak at 1542 cm^–1^ could be related to the skeletal vibration of graphene.^60,61^

### 3.2 Roughness and Transmittance

**Figure 4:**
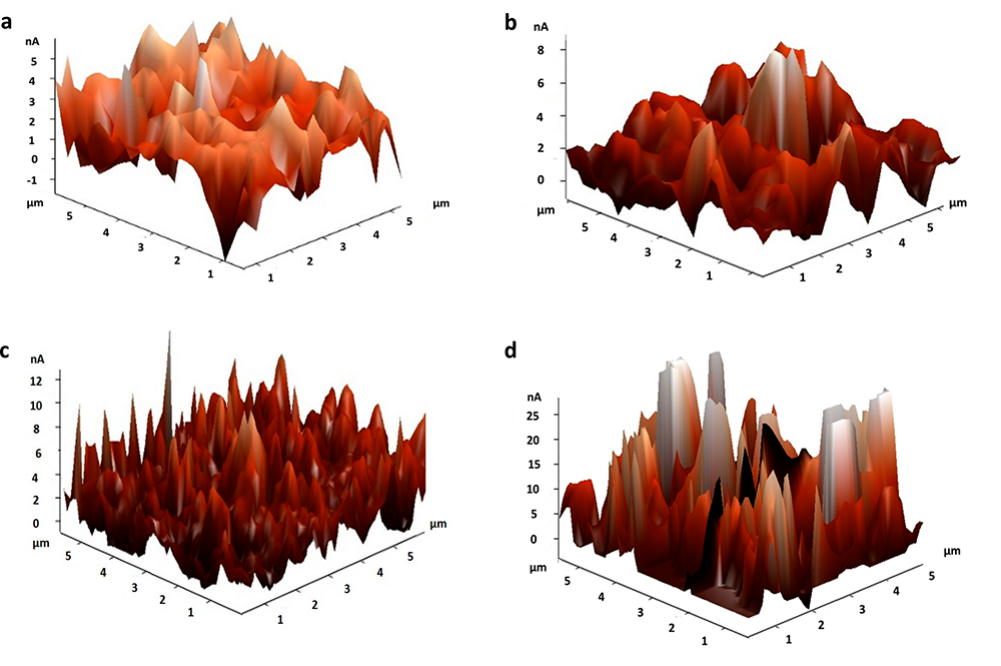
AFM images of (a) TiO_2_, (b) TiO_2_@Si, (c) TiO_2_@Si:Fe, and (d) TiO_2_@Si:Fe:rGO coated thin films.

Figure 4 shows the surface topography of the coated films investigated by AFM. The AFM results imply that the average roughness (*R*_a_) of the films coated with TiO_2_, TiO_2_@Si, TiO_2_@Si:Fe, and TiO_2_@Si:Fe:rGO are 40, 47, 69, and 200 nm, respectively. As can be seen, *R*_a_ has a slight increase with the addition of SiO_2_ and γ-Fe_2_O_3_ and a sharp increase with the addition of rGO.^62,63^ This result could be correlated to sloppy stacking of rGO sheets, which results in larger channels around the rGO stacks, compatible with the variation in surface roughness.^64^ In other words, the rGO assembled layers in TiO_2_@Si:Fe:rGO coating probably are in random order and, therefore, are very rough on the surface.^65^

As highlighted above, only such protective coatings which satisfy the maintaining of the aesthetic appearance of artifacts through a transparent and colorless layer can be acceptable.^1,66^ Hence, coatings producing an apparent color difference with the substrate are not appropriate for metal protection from discoloration in modern and ancient artifacts, analogously to pointing already drawn in the case of glass both in the solar cell field and in the built environment.^67^

Transmittance spectra in the range of 300–1000 nm were measured on glass slides coated with the different coating materials. As can be seen in Table 4, all coated layers have high transmittance over the entire visible wavelengths range, higher than 80% also in the case of the most complex TiO_2_@Si:Fe:rGO coating. This proves that all coatings have good transparency.

**Table 4:**
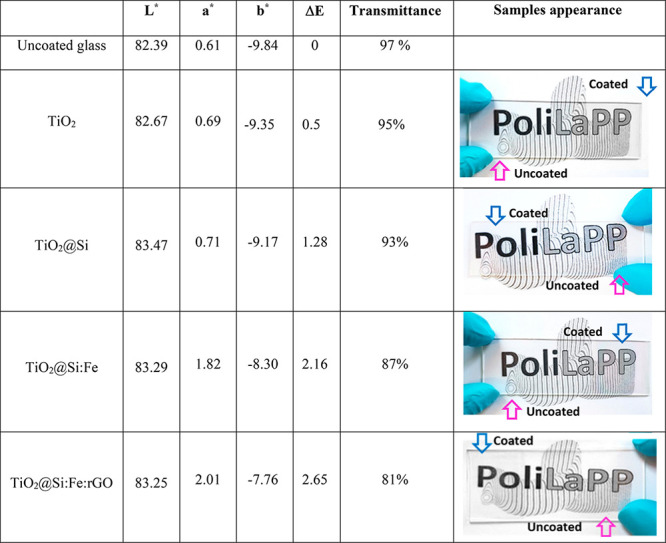
L*, a*, and b* Colorimetric Coordinates, Color Change Δ*E*, and Transmittance for Uncoated and Coated Glasses with TiO_2_, TiO_2_@Si, TiO_2_@Si:Fe, and TiO_2_@Si:Fe:rGO Thin Films

Although transmittance slightly decreased with the addition of SiO_2_, γ-Fe_2_O_3_, and rGO, all samples were still satisfactory from an aesthetic viewpoint. The increased surface roughness could be responsible for the slight loss in optical transmission in the TiO_2_@Si:Fe:rGO coating (see Figure 4). Furthermore, TiO_2_@Si:Fe film was observed to have a higher absorption intensity than TiO_2_@Si (Figure S3). This issue could be a result of electronic interactions between TiO_2_@Si and γ-Fe_2_O_3_ and efficient surface hybridization between these components. Moreover, the TiO_2_@Si:Fe:rGO coated film exhibits the highest absorption compared to the other samples, which is expected due to the large optical absorption of both γ-Fe_2_O_3_ and rGO. None of these coatings generated a color perceivable by naked eyes easily. Spectrophotometric measurements in reflectance mode were performed as well to observe color differences with more details (Table 4). These measurements were done by placing the glass slide on a reference white surface before and after dip coating, to evaluate color changes induced by the application of the coating.

Table 4 also reports the average L*,a*,and b* color coordinates for uncoated and coated samples and the Δ*E* values calculated using the uncoated sample as reference. In all specimens, the coating induced a slight whitening, observable as a small increase in the luminance parameter (L*) in TiO_2_ and TiO_2_@Si. However, L* decreased again for samples TiO_2_@Si:Fe and TiO_2_@Si:Fe:rGO, while still higher than uncoated glass. The a* parameter increased in TiO_2_@Si:Fe and TiO_2_@Si:Fe:rGO samples as a result of the inherent red color brought about by γ-Fe_2_O_3_. Also, the b* coordinate slightly increased with the application of the coating, indicating a small reduction in the blue hue intensity. The values of the overall color difference Δ*E* (Table 4) indicate that the TiO_2_ coating had no effect on the aesthetic appearance of the specimen with Δ*E* < 1, meaning not perceptible by human eyes. However, by adding other components, particularly γ-Fe_2_O_3_, Δ*E* values increased slightly, but still below 3, which means the color differences are perceptible only by close observation and are therefore more than acceptable.

### 3.3 Photoactivity under UV and Visible Lights

**Figure 5:**
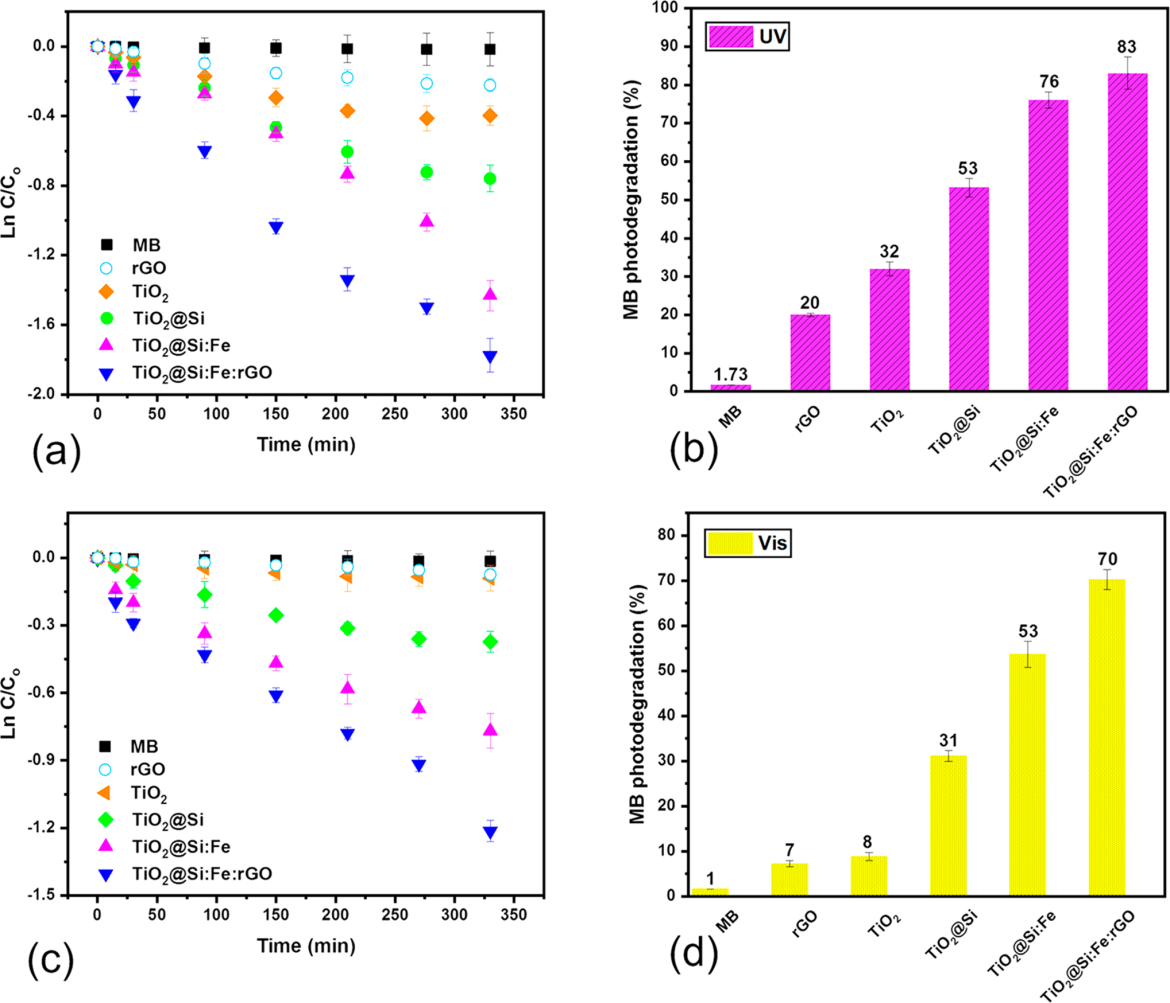
Photocatalytic activity for degradation of MB under (a,b) UV and (c,d) vis lights for MB, TiO_2_, TiO_2_@Si, TiO_2_@Si:Fe, and TiO_2_@Si:Fe:rGO coated films.

The photocatalytic degradation of MB with TiO_2_, rGO, TiO_2_@Si, TiO_2_@Si:Fe, and TiO_2_@Si:Fe:rGO coated films under UV and visible lights was investigated (Figure 5). First, samples were immersed in the dye solution in dark: the equilibrium adsorption state was reached in 30 min, and MB adsorption on all of the samples was negligible. Degradation extents after 6 h UV irradiation were about 32% for pure TiO_2_, 20% for pure rGO, 54% for TiO_2_@Si, and more than 76% and 83% for TiO_2_@Si:Fe and TiO_2_@Si:Fe:rGO, respectively. These amounts decreased to about 9%, 8%, 32%, 54%, and 71% under visible light.

Results were plotted as ln *C*/*C*_0_ versus time, obtaining linear trends with correlation coefficients (*R*^2^) higher than 0.9 as expected in the pseudo-first-order kinetics model.^55,64^ Kinetic constant (*k*) and band gaps of TiO_2_, TiO_2_@Si, TiO_2_@Si:Fe, and TiO_2_@Si:Fe:rGO coated films are reported in Table 5. The direct band gap values reported were determined by extrapolating the linear region of the Tauc’s plot of (α*h*υ)^0.5^ against photon energy, starting from absorbance data reported in Figure S3.

**Table 5:** Kinetic Constants of Photocatalytic MB Degradation under UV and Visible Light and Band Gaps of TiO_2_, rGO, TiO_2_@Si, TiO_2_@Si:Fe, and TiO_2_@Si:Fe:rGO Thin Films^a^

	UV		visible		
sample	*K* (min^–1^)	*R*^2^	*K* (min^–1^)	*R*^2^	band gap (eV)
MB	n/a	0.95	n/a	0.93	
rGO	n/a	0.94	n/a	0.95	
TiO_2_	–0.0013	0.93	n/a	0.91	3.20
TiO_2_@Si	–0.0024	0.98	–0.0012	0.94	3.03
TiO_2_@Si:Fe	–0.0040	0.97	–0.0022	0.97	2.87
TiO_2_@Si:Fe:rGO	–0.0053	0.98	–0.0032	0.98	2.26

a n/a = too low to be evaluated.

The increased efficiencies obtained in each addition of a compound to the sol are explained in the following. SiO_2_ both increased the anatase content and reduced particle size, avoiding agglomeration. γ-Fe_2_O_3_ improved charge separation and reduced electron–hole recombination by receiving in its conduction band (CB) electrons photogenerated in TiO_2_ and transferring holes photogenerated in its valence band (VB) to that of TiO_2_. The incorporation of rGO into the composite led to band gap reduction. In fact, as shown in Figure S3 light absorption increased in the TiO_2_@Si:Fe:rGO nanocomposite, suggesting that this sample could exhibit an enhanced photocatalytic activity. Moreover, photogenerated electrons can easily migrate to the rGO sheets, leading to effective separation and decreased recombination rate of photogenerated electron and hole pairs. In fact, the sheets of rGO can act as an excellent speed charge transfer channel to enhance the charge separation efficiency. Also, rGO sheets keep nanoparticles dispersed, preventing agglomeration.^68−71^

### 3.4 Evaluation of Coating Integrity

**Figure 6:**
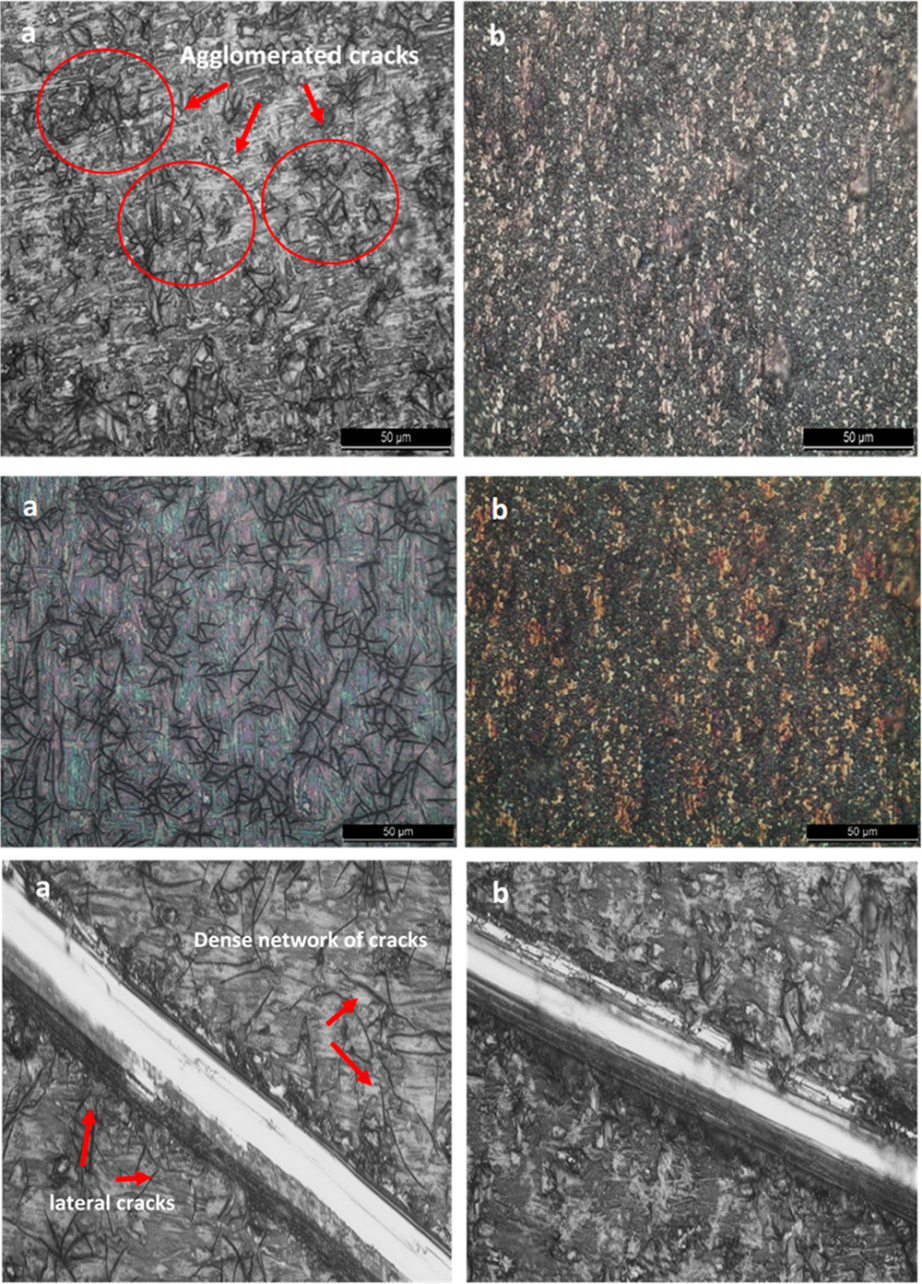
Optical microscopy images (500×) of newly deposited thin films (top), same coatings after acid rain test (middle), and scratched surfaces (bottom) (scale bars represent 25 μm): (a) TiO_2_ and (b) TiO_2_@Si:Fe:rGO films.

As mentioned above, mechanical durability and excellent adhesion of coatings could guarantee them undamaged in long-term use in cultural heritage field. We compared durability and surface structure of the TiO_2_@Si:Fe:rGO and TiO_2_ coated films by OM analysis (Figure 6). While TiO_2_ presents several cracks on its surface, the TiO_2_@Si:Fe:rGO coated film presents a uniform and dense structure without cracks. Surface structure was evaluated even after long-time immersion and photocatalysis tests. While the TiO_2_@Si:Fe:rGO film remains unchanged during degradation tests, the amount of cracks in the TiO_2_ coating increased significantly after exposure to the acid environment due to its weak stability.

Scratch test was performed as a destructive method to test the adhesion of such soft coatings deposited on hard substrates such as titanium^72^ (Figure 6). As revealed by OM images, in the TiO_2_ film, dense networks of lateral cracks could be observed alongside the scratch trail on the surface. These cracks are much more apparent compared to the TiO_2_@Si:Fe:rGO film, with considerably fewer nucleation regions to cracking; in the latter case, by rubbing the scratched region with fingers, the scratched film was not easily removed.

Generally, the improvement in interfacial adhesion and mechanical stability could be related to better interactions between the TiO_2_@Si:Fe:rGO film and titanium surface. According to previous reports, the addition of SiO_2_ and Fe_2_O_3_ to TiO_2_ thin films could display very high adhesive and cohesive strengths in comparison with pure TiO_2_ thin films.^73−78^ Besides, the unique two-dimensional carbon-based structures such as GO (and rGO) sheets could act effectively at deflecting cracks at the metal/film interface, also improving coating density and adhesion through a multilayer structure.^79−84^

### 3.5 Self-Cleaning and Discoloration Resistance Tests

**Figure 7:**
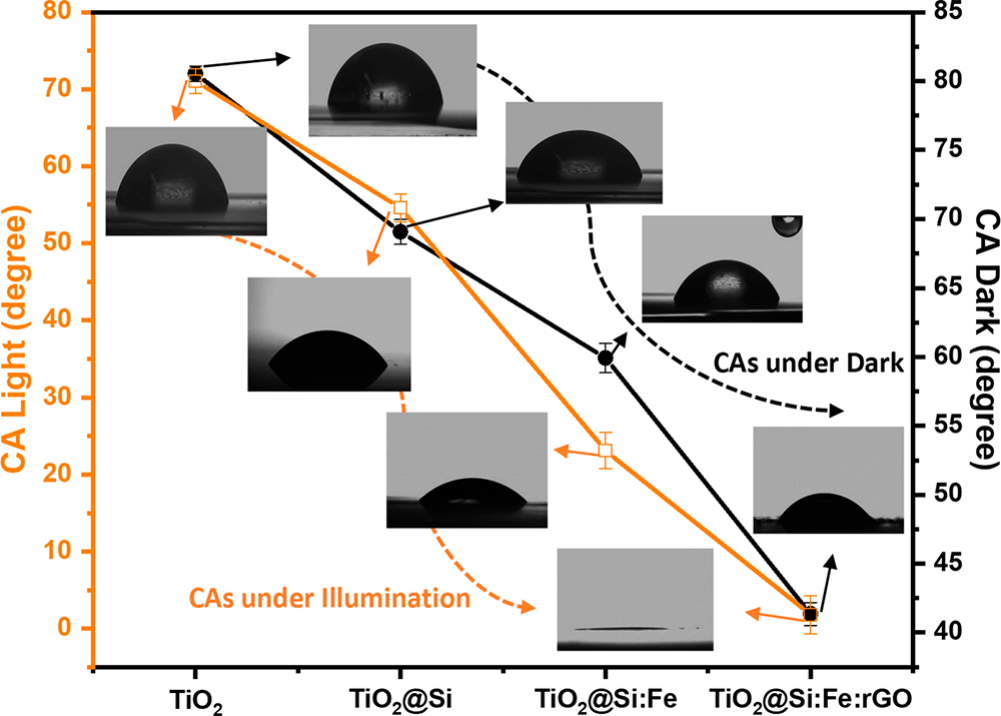
Evolution of water CA in dark and under illumination, with images of water CAs on TiO_2_, TiO_2_@Si, TiO_2_@Si: Fe, and TiO_2_@Si: Fe:rGO.

Wettability, measured by water contact angle (CA), is a critical property in self-cleaning studies depending on the chemical composition and the morphology of the solid surfaces.^85−87^ The surface wettability of the coated surfaces was investigated by measuring CA in the dark and after light irradiation for 30 min. The shape of the water droplet and CA values are given in Figure 7.

CA values measured before and after light irradiation show significant differences between coated substrates. In particular, water CA on TiO_2_@Si coating decreases after exposure to light, probably due to SiO_2_, which suppresses the transformation of anatase to rutile and the crystal growth of anatase (see Figure 2a,b). Furthermore, it favors water adsorption as shown by increased surface hydroxyl groups observed in FTIR characterization, which helps superhydrophilicity be maintained for an extended period.^88^

Clearly, by adding γ-Fe_2_O_3_ to TiO_2_/SiO_2_ structure, wettability under light irradiation further increased thanks to the improved charge separation and decreased recombination phenomena.^89^ Indeed, the introduction of Fe^3+^ ions in TiO_2_@Si:Fe film is responsible for a reduction in the photogenerated hole–electron recombination rate. Then, more photogenerated holes can oxidize the lattice O_2_^–^ anions, creating more oxygen vacancies; eventually, water molecules may coordinate into the oxygen vacancy sites, leading to dissociative adsorption of water molecules on the surface, increasing surface hydroxyl content and reducing CA upon irradiation.^90^

The decrease in CA appeared much more abrupt by adding rGO, both in the dark and under light. Indeed, rGO had a major effect on wettability: after illumination, water CA changed clearly from hydrophilic (CA 41.3°) to superhydrophilic with CA < 2°. Furthermore, CA remained relatively constant even after turning off light and approached the initial dark state gradually.

The role of rGO on coating wettability could be attributed to the following mechanism. With the aid of UV irradiation, oxygen molecules on graphene are motivated from the ground spin-triplet state to a higher energy spin-singlet, resulting in electron–hole pairs which in turn form superoxide anions.^91,92^ This leads to depletion of a large number of oxygen atoms, which in turn results in a continuous diffusion of oxygen vacancies on the rGO surface. Water molecules in air capture these vacancies to generate hydroxyl radicals, which bind to the carbon atom of the rGO in a supramolecular form (rather than in the form of a covalent bond) converting the hydrophilic TiO_2_@Si:Fe:rGO composite (in the dark) into superhydrophilic TiO_2_@Si:Fe:rGO under irradiation.^93,94^ The adsorption of water molecules on the rGO defect surface further imparts it with superhydrophilicity. Under dark, these supramolecules gradually disappear on account of a lack of adequate energy supply.^94^ Subsequently, oxygen molecules substitute for water molecules deposited on the graphene defect surface, which induces the return to hydrophilicity. If hydroxyl radicals were attached to the carbon atom of rGO by covalent bonds, they would not disappear in the absence of light. In the case of rGO, the hydroxyl groups are covalently bound to carbon atoms, so after a time in the dark storage, the rGO surface is not capable of converting into the initial CA immediately because of their presence. The following equations can be used to describe the reaction mechanisms:
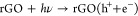
2
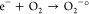
3
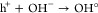
4
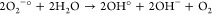
5

In addition to the above discussions, transition in CA can be related to surface properties, primarily surface roughness. As a result, the droplet can spread on uneven surfaces formed after adding the components SiO_2_, γ-Fe_2_O_3_, and rGO, as indicated by the increasing surface roughness (Figure 4), and thus enhancing hydrophilicity.^95,96^

**Figure 8:**
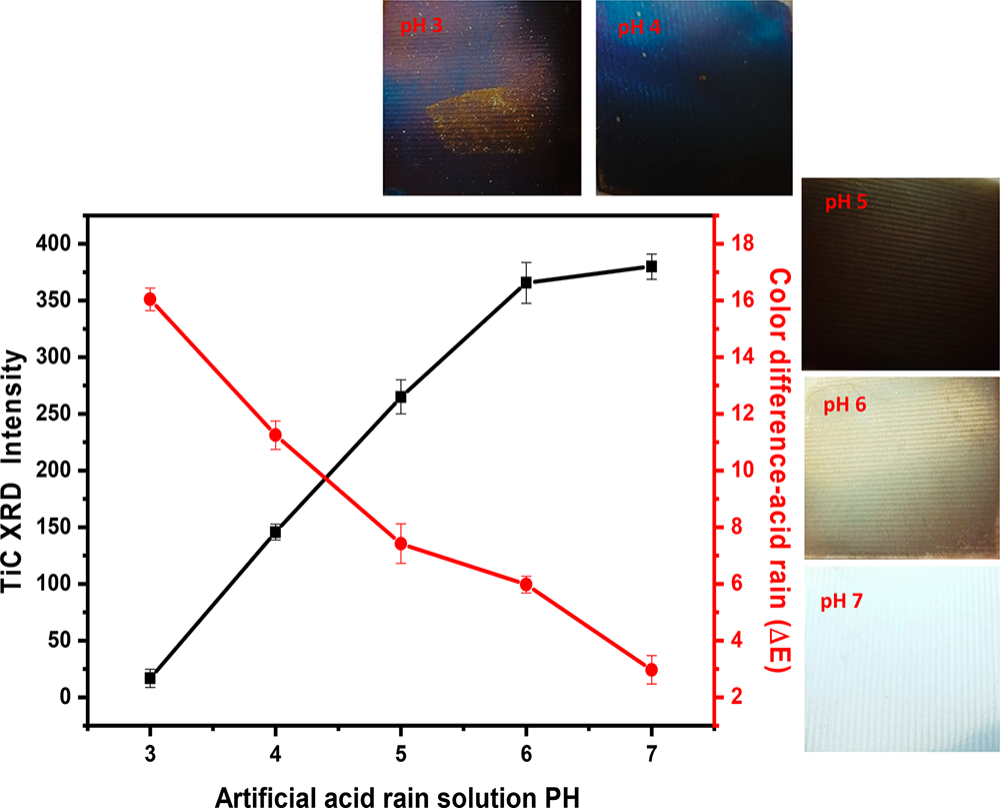
Effect of different pHs on the discoloration of titanium sheet and TiC XRD intensity (accelerated discoloration test: 65 °C, 7 days).

Figure 8 shows the effect of different artificial acid rain solutions of pHs 3–7 on color differences and the amount of TiC (detected by XRD) on bare uncoated specimens after immersion for 7 days (also see Figures S4 and S5 and Table S1). As the pH fell to roughly 4.5 or below (acidic), color alteration, Δ*E*, increased rapidly, leading to discoloration comparable to that in the outdoor atmosphere. The color difference, Δ*E*, increased with immersion time, exceeding a value of 16 with an artificial acid rain of pH 3. However, in the pH 7 solution color variations were lower than 3, hence not noticeable.

It is presumed that discoloration in the atmosphere is an interference color caused by the dissolution of titanium in acid rain, which forms TiO_2_ on the sheet surfaces; the oxide film grows thicker, causing the interference color when thickness exceeds a few tens of nanometers.^4^ The presence of TiC can also play a role in discoloration, as underlined by the following considerations. When discoloration accelerates markedly, the presence of TiC on the surfaces decreases significantly (see Figure 8). Indeed, TiC dissolves more than ten times more easily in a sulfuric acid solution than metallic titanium does.^37^ The dissolved TiC quickly forms Ti ions, which then turn into TiO_2_ through hydrolysis, depositing on the metal surface and increasing discoloration by interference as the oxide film grows.^37^

**Figure 9:**
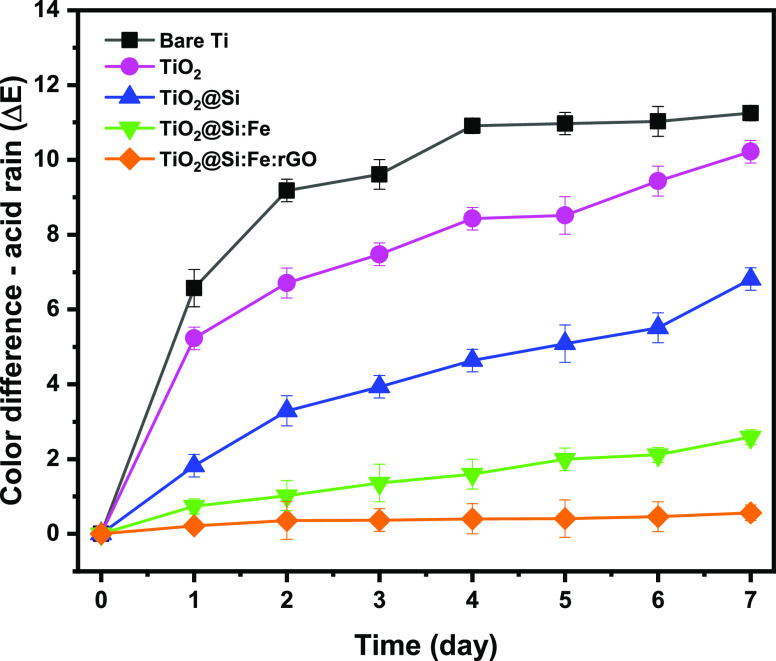
Discoloration test results of bare Ti and coated Ti with TiO_2_, TiO_2_@Si, TiO_2_@Si:Fe, and TiO_2_@Si:Fe:rGO sols in an acid solution of pH 4 at 65 °C.

In contrast with other samples, at pH 4—where bare titanium already suffered from a heavy attack—in TiO_2_@Si:Fe:rGO coated specimens, the color difference was less than 1 and not distinguishable by naked eyes, indicating that the coated film protects the substrate significantly with barrier effect (Figure 9).

Despite excellent discoloration resistance of the TiO_2_@Si:Fe:rGO coated specimens (and considerably the TiO_2_@Si:Fe film), other samples are susceptible to degradation at pH 4. Namely, the color difference of TiO_2_ and TiO_2_@Si sheets increased with immersion time in acid rain, reaching values of 7 and 10, respectively.

Furthermore, results in Figure S6 and Table S2 demonstrate that the wettability of the TiO_2_@Si:Fe:rGO thin film is not affected significantly by the discoloration tests. Interestingly, the film not only could protect titanium from discoloration but also kept its own integrity against chemical or physical decomposition, which could result in crack formation. In contrast, TiO_2_ wettability (as a comparison) showed an increase by about 10° to 15° both in dark and under illumination after discoloration. As the TiO_2_ film could not prevent titanium from changing color, i.e., from oxidizing (see Figure 9), then the new oxide film growth may have damaged the sol–gel derived TiO_2_ film.^97^ Consequently, this led to a change in wettability, tending more to hydrophobicity.

**Figure 10:**
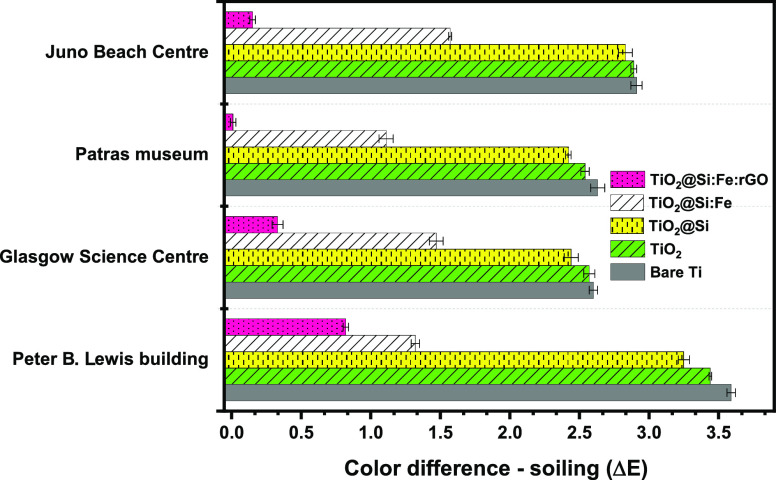
Color difference due to the simulated soiling test for the bare and coated titanium specimens exposed to soiling conditions simulating different places (June beach center (France), Patras museum (Greece), Glasgow science center (The U.K.), and Peter B Lewis building (USA)).

Figure 10 shows the color difference developed in simulated soiling tests for uncoated and coated titanium specimens exposed to the five different soiling conditions simulating the different environments considered. As can be seen, the self-cleaning performance of the specimens depends on the type of pollutants, which are different on the basis of the place. As expected, all of the coated sheets show lower Δ*E* than the bare sheet, while coated films do not show similar behaviors. On the basis of the results, it can be discussed that TiO_2_@Si:Fe:rGO coated film could act more efficiently rather than other films thanks to its significant self-cleaning properties. This result was expected considering its superhydrophilicity and excellent photoactivity after illumination, as discussed above. Furthermore, results (Figure S7 and Table S3) confirm that the TiO_2_@Si:Fe:rGO film could keep its appearance almost unchanged even after the soiling tests.

**Figure 11:**
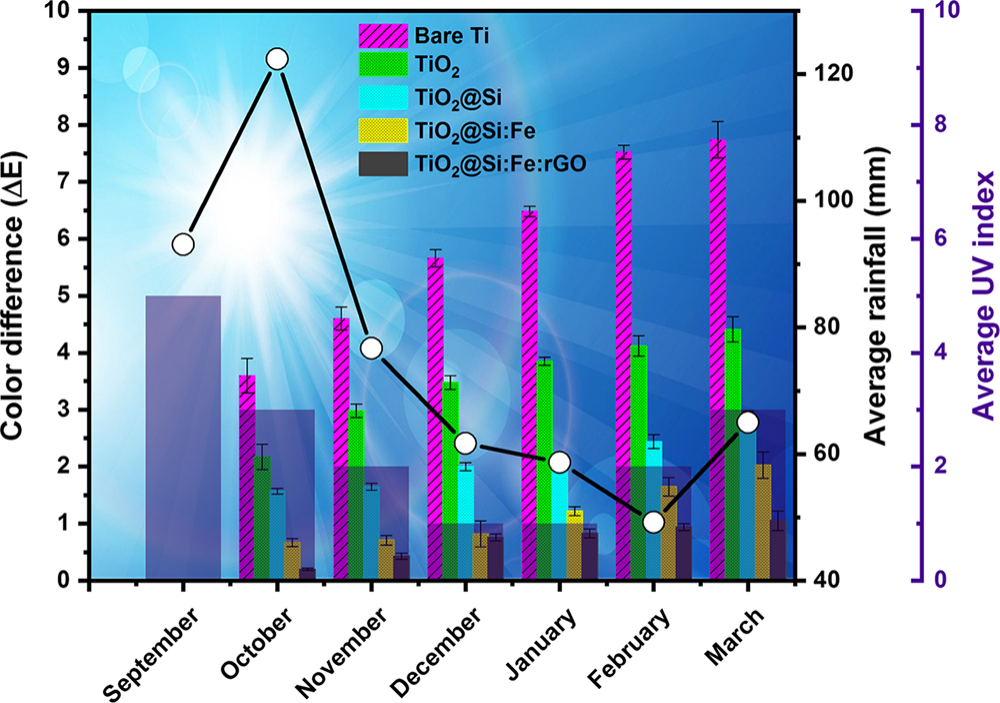
Color difference of the bare and coated titanium specimens exposed to the roof for 6 months (affected by average rainfall and UV index).

Soiling in real outdoor conditions was actually performed by exposing samples to the polluted atmosphere of Milan, Italy. Figure 11 shows Δ*E* values measured at the beginning of each month during the six months exposure. Moreover, the local average rainfall and sunlight irradiation index obtained from “air.plumelabs“ and “weather-atlas” databases during this period are indexed in the graph.

The high pollution concentration in September (41.2 μg/m^3^) (Table 6) gave rise to an initial steep increase of Δ*E* values for all samples, coated and uncoated. As expected, bare titanium showed higher Δ*E* due to the lack of any protective self-cleaning layer on the surface. Interestingly, in the case of the TiO_2_@Si and TiO_2_@Si:Fe films, the coated layers preserved the titanium sheet by on setting self-cleaning conditions, benefiting from considerable rainfall and UV irradiation. Afterward, color-changing increased unfavorably, especially on bare titanium and TiO_2_ coated substrates. In the case of TiO_2_@Si and TiO_2_@Si:Fe films, these changes were more significant compared to the contribution of the first month, due to low related rainfall and UV which decreased the chances of self-cleaning.

**Table 6:** Pollutant Concentration (μg/m^3^) from September to March

	Sep	Oct	Nov	Dec	Jan	Feb	Mar
pollutant concn (μg/m^3^)	41.2	19.5	23	16.5	50	49	18

The most noticeable results were obtained once again on the TiO_2_@Si:Fe:rGO coated film after six months of exposure, with an overall Δ*E* lower than 1, meaning that the appearance did not show any color difference detectable by eye, meaning that it maintained self-cleaning properties also in a period of low rainfall and UV.

## 4 Conclusion

The fabricated transparent TiO_2_@Si:Fe:rGO composite showed excellent photoactivity and wettability, behaving well in self-cleaning applications. Addition of SiO_2_ improves the morphology, crystalline structure, and surface hydroxylation of TiO_2_ nanoparticles and γ-Fe_2_O_3_ decreases the recombination rate of e^–^/h^+^ pairs and largely improves photocatalytic activity under visible light. Moreover, the rGO sheets also reduce recombination and improve nanoparticle dispersion. rGO also showed a significant effect on wettability, leading to a lower contact angle of the TiO_2_@Si:Fe:rGO coated surface in dark and to superhydrophilicity under irradiation. On the basis of this study, the prepared TiO_2_@Si:Fe:rGO composite coating is an efficient photocatalyst for degradation of contaminants under UV and visible light illumination and allows one to convert surface wettability to superhydrophilicity. Furthermore, the film revealed a proper adhesion integrity even after photodegradation tests, as well as superhydrophilicity even after discoloration tests.

Concerning the self-cleaning behavior, all coatings were sufficiently transparent to avoid alteration of the substrate color. Moreover, simulation of contact with acid rain caused negligible color change for TiO_2_@Si:Fe:rGO coated surface. On the basis of simulated soiling tests, the same coating allowed efficient self-cleaning thanks to its significant superhydrophilicity and excellent photoactivity. These results were confirmed by roofing tests, where again negligible color changes were induced by atmospheric exposure of the coated samples. These results pave the way for potential use of the produced coating as a protective layer on metallic substrates working outdoors, such as in the built environment.

## Supporting Information Available

Synthesis schematic representation, artifacts photographs, samples’ absorbance spectra, discoloration photographs, samples’ color differences, XRD results, contact angle photographs, and photographs of samples after soiling tests (PDF)
