# Supplementary material for: Heterostructured TiO2/SiO2/γ-Fe2O3/rGO Coating with Highly Efficient Visible-Light-Induced Self-Cleaning Properties for Metallic Artifacts
Source: ACS Appl Mater Interfaces. 2020 Jun 3;12(26):29671–83. doi: 10.1021/acsami.0c06792 (PMC8153401; doi:10.1021/acsami.0c06792)
Supplement: Supplementary file 1 — am0c06792_si_001.pdf [file am0c06792_si_001.pdf]

Supporting Information

Heterostructured $TiO_2/SiO_2/\gamma$-Fe_2O_3/rGO coating with highly efficient visible-light induced self-cleaning properties for metallic artifacts

Maryam Mokhtarifar [1], Reyhaneh Kaveh [2], Mojtaba Bagherzadeh [2], Andrea Lucotti [1], MariaPia Pedeferri [1], and Maria Vittoria Diamanti [*1]

[1] Dept. of Chemistry, Materials and Chemical Engineering "Giulio Natta", Politecnico di Milano, Milan, Italy

[2] Dept. of Chemistry, Sharif University of Technology, Tehran, Iran

*corresponding author email: mariavittoria.diamanti@polimi.it

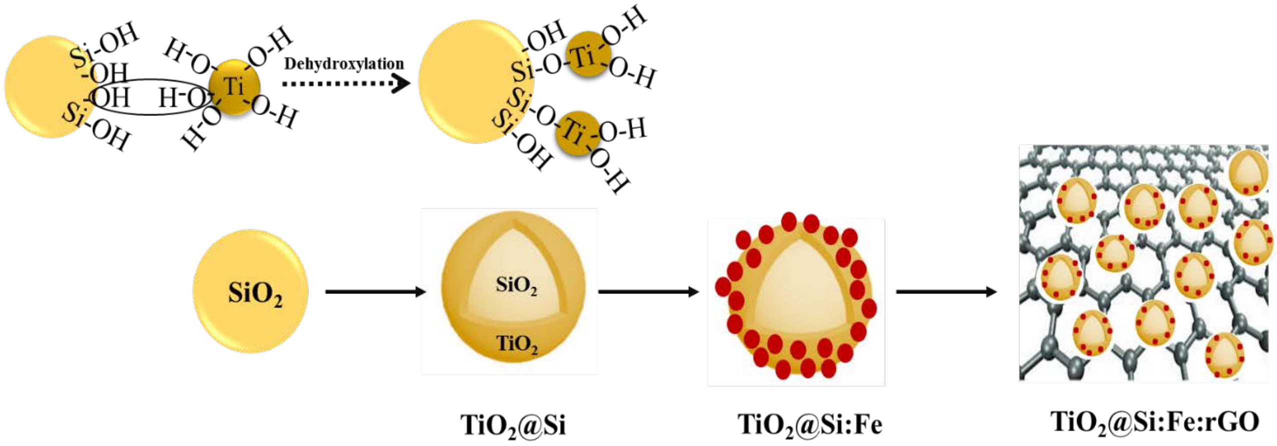

Figure S1: Schematic representation of the TiO_2@Si:Fe:rGO synthesis.

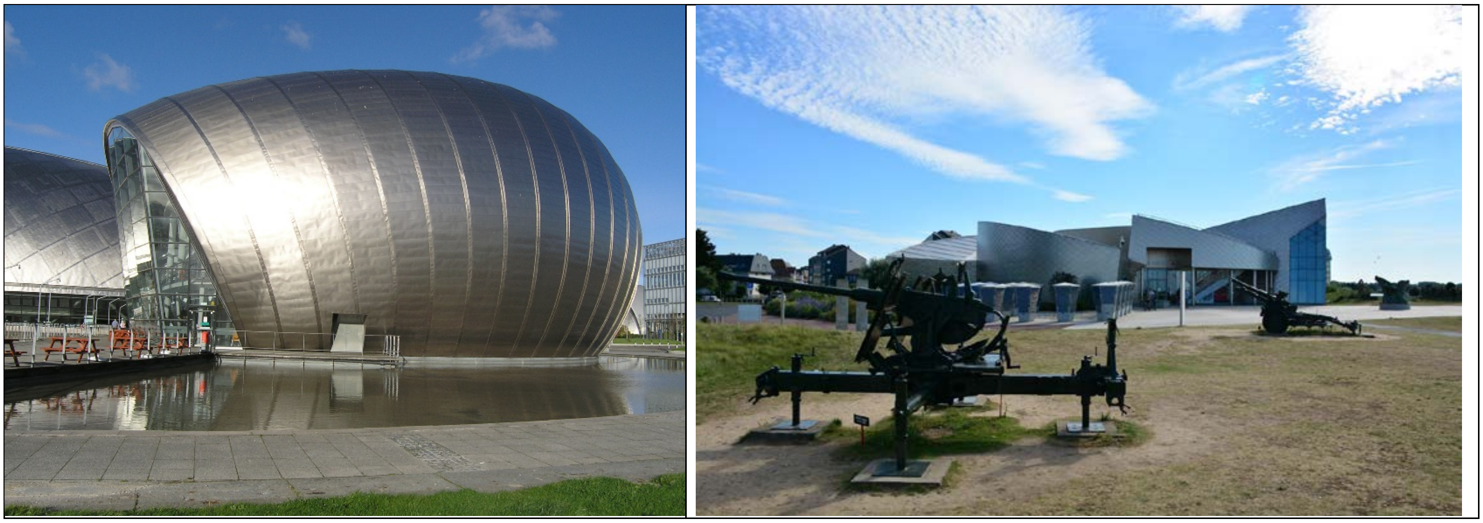

Figure S2. Two of the modern and contemporary titanium-based artifacts considered in this study for simulated soiling: left, Glasgow Science Centre, Glasgow, United Kingdom (picture by Kanakari, CC license https://creativecommons.org/licenses/by-sa/3.0/deed.en; no changes were made), and right, Juno Beach Centre, Normandy, France (picture by Paul Arps, CC license https://creativecommons.org/licenses/by/2.0/; no changes were made).

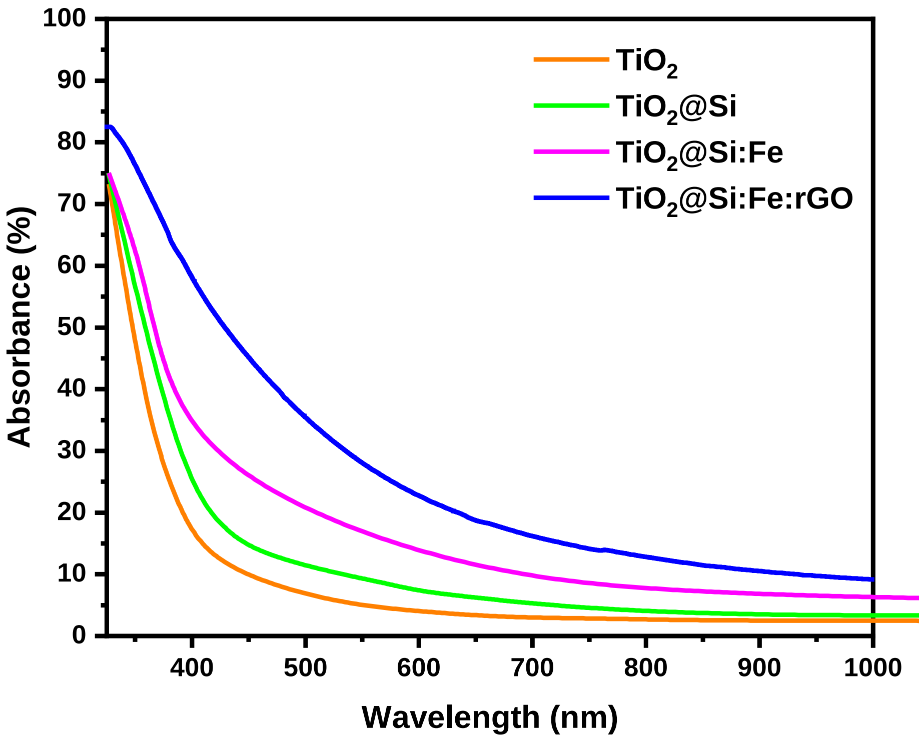

Figure S3. Absorbance spectra of TiO_2, TiO_2@Si, TiO_2@Si:Fe, and TiO_2@Si:Fe:rGO.

Table S1. Color difference of bare Ti specimens after 7 days exposure to the solution with different pH from 3 to 7

Days	pH 3	pH 4	pH 5	pH 6	pH 7
0	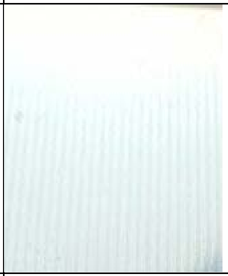	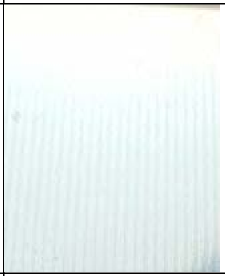	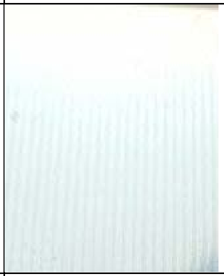	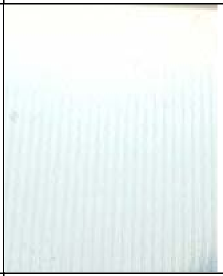	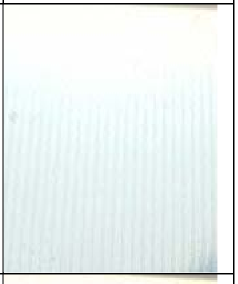
3	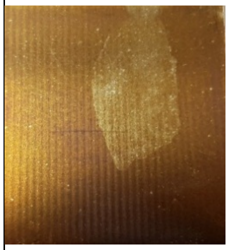	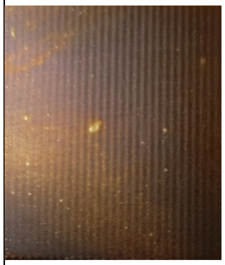	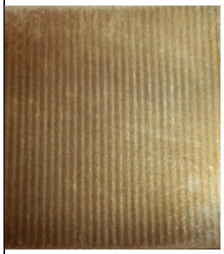	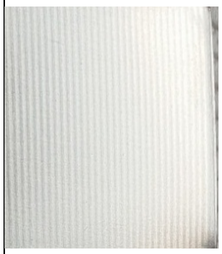	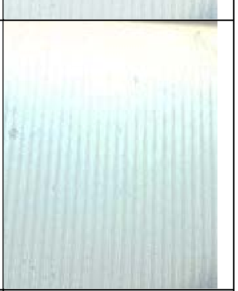
4	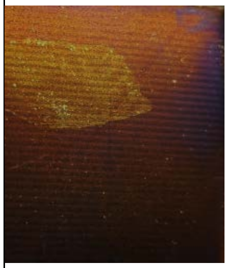	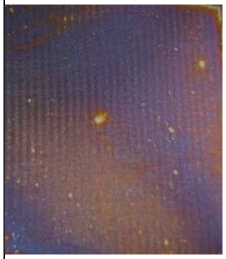	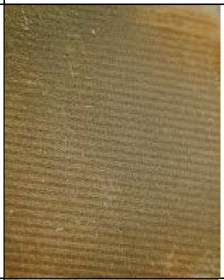	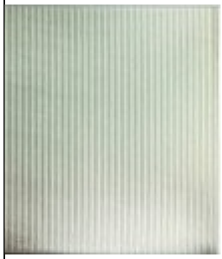	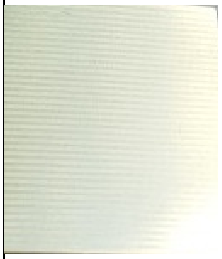
5	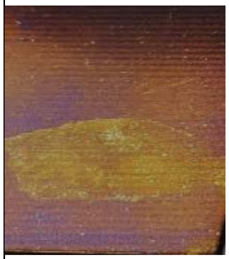	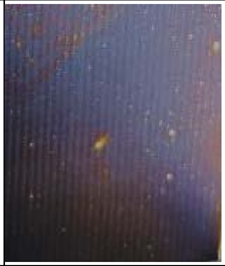	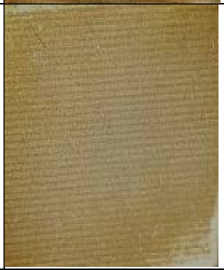	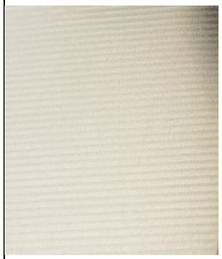	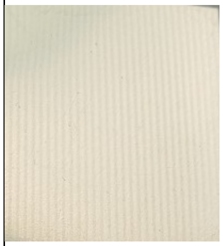
6	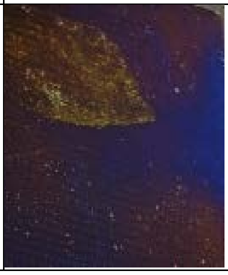	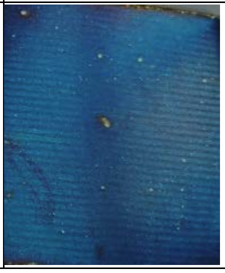	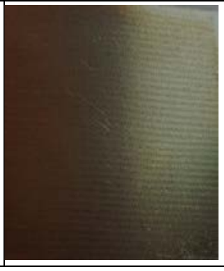	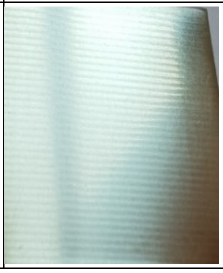	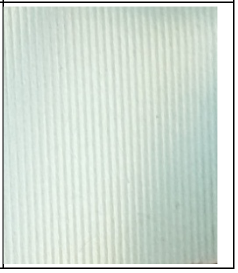

7	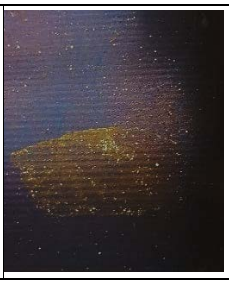	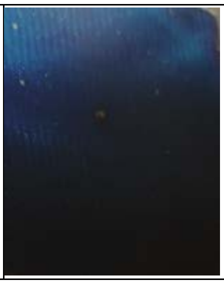	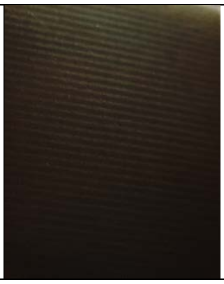	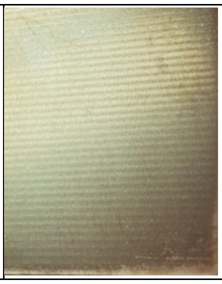	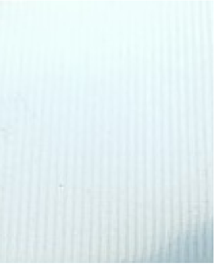

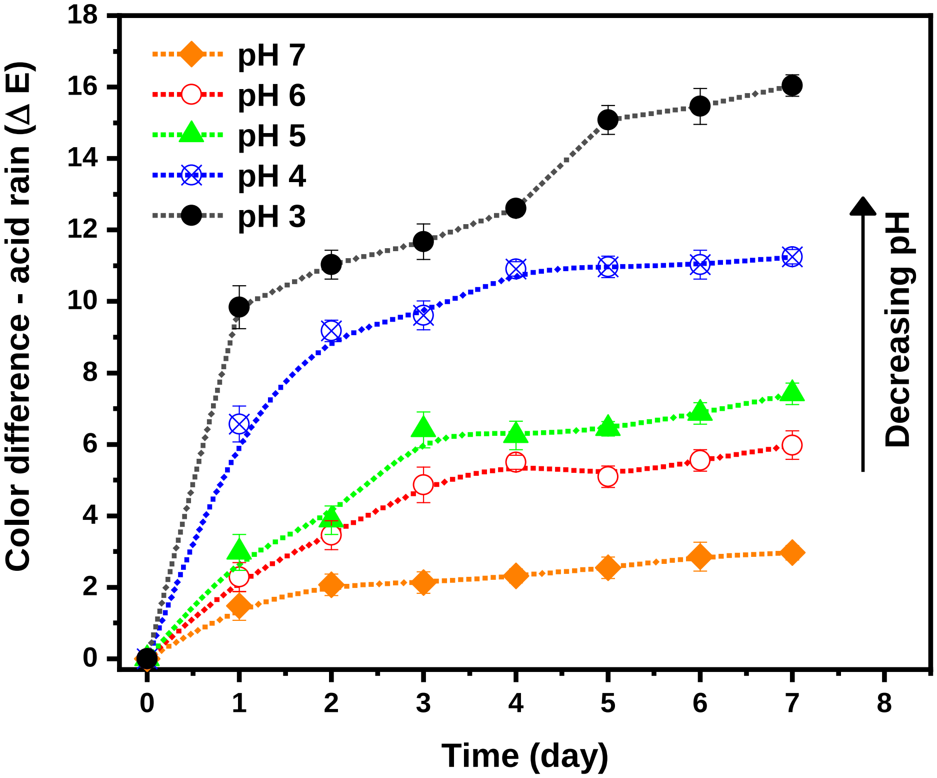

Figure S4. Effect of different pHs on the discoloration of titanium sheet (accelerated discoloration test: 65˚C, 7 days).

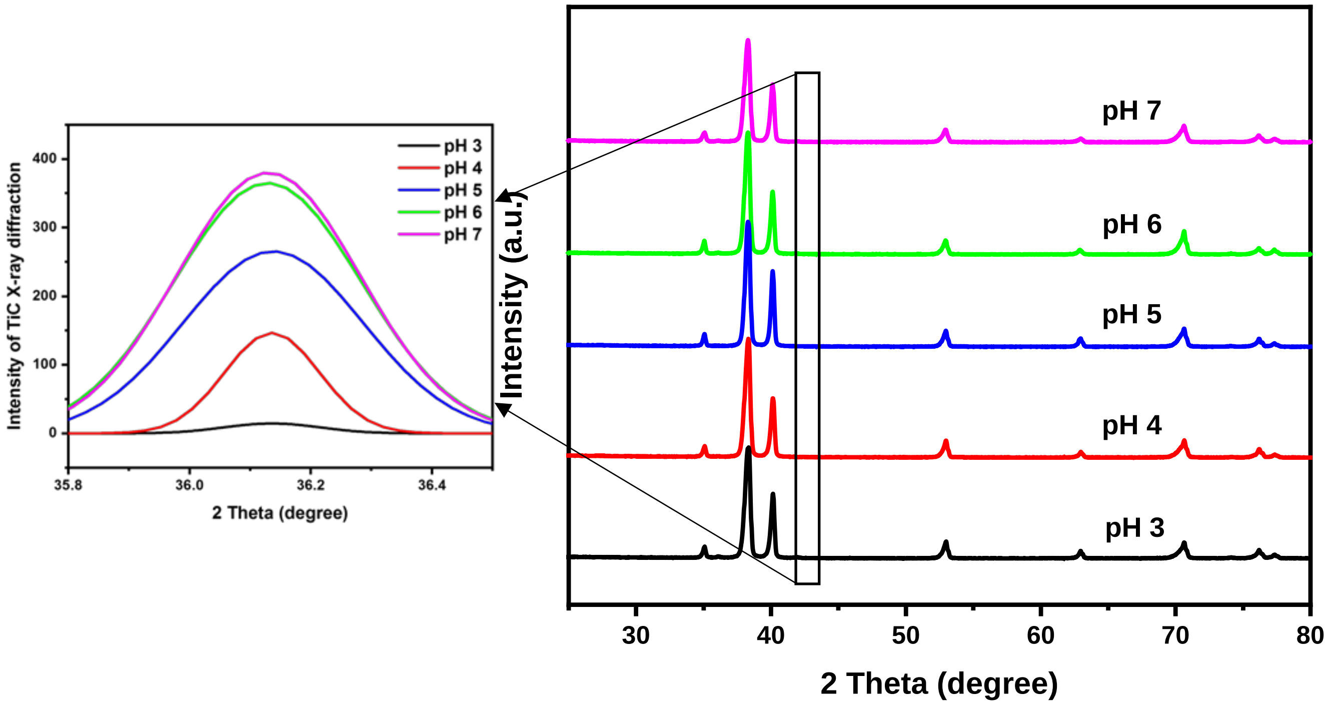

Figure S5. XRD results for bare Ti specimens after 7 days of exposure to the solution with different pH from 3 to 7.

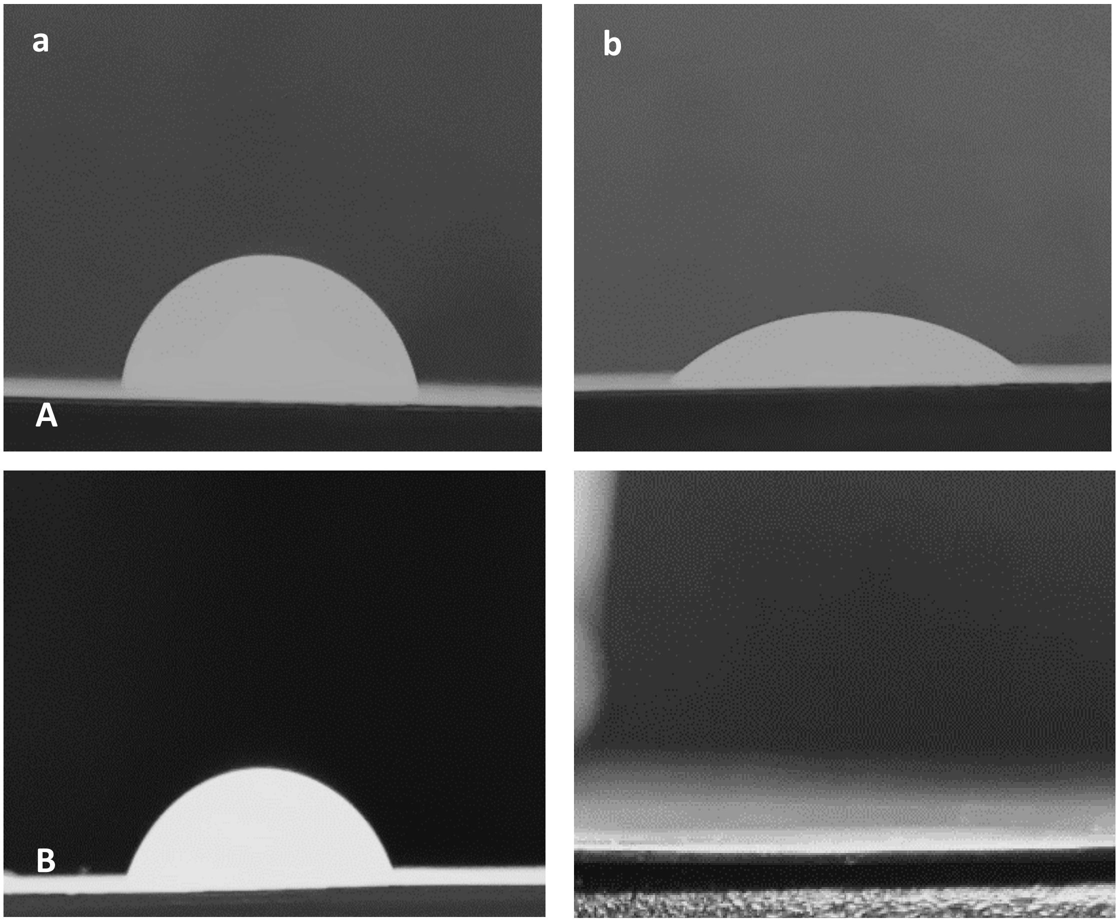

Figure S6. The contact angle after simulated acid rain tests in dark (A) and under illumination (B), a) TiO_2 and b) TiO_2@Si:Fe:rGO coated films.

Table S2. Contact angle before and after simulated acid rain tests in dark and under illumination for TiO_2 and TiO_2@Si:Fe:rGO coated films

	CA Dark Before the acid rain test	CA Light Before the acid rain test	CA Dark After the acid rain test	CA Light After the acid rain test
TiO_2	79	70	90	84
TiO_2@Si:Fe:rGO	41.3	1.9	45.2	2.7

Figure S7. The transparency of TiO_2@Si:Fe:rGO coated film after soiling test, a) Peter B lewis building (the USA), b) Glasgow science centre (The UK), c) June beach center (France), and d) Patras museum (Greece).

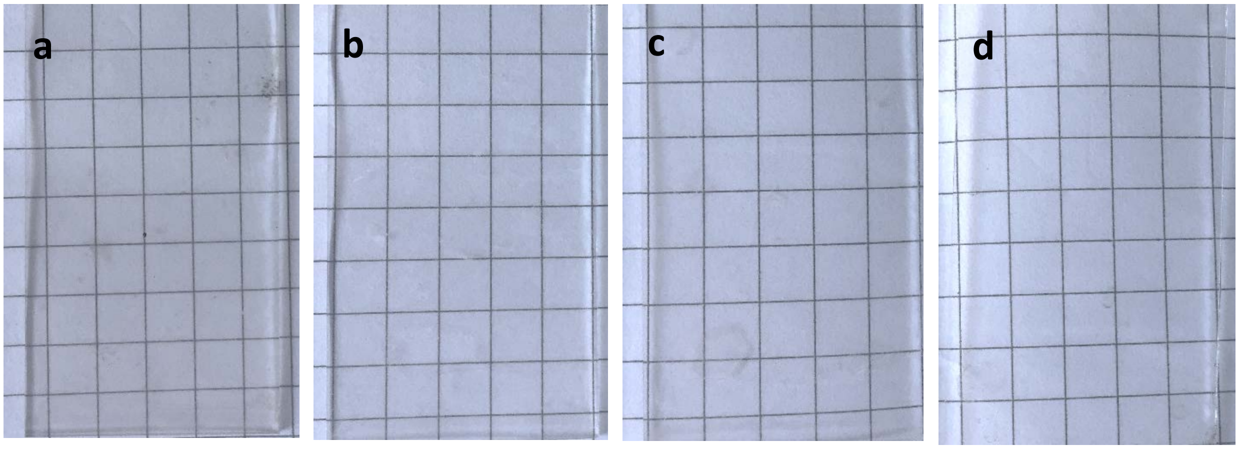

Table S3. Color change (ΔE) of uncoated glass and glass coated with TiO_2@Si:Fe:rGO thin film after soiling

ΔE	Peter B lewis building	Glasgow science centre	June beach center	Patras museum
Unsoiled TiO_2@Si:Fe:rGO coated glass	0.62	0.46	0.49	0.38
Bare glass	3.11	2.87	2.73	2.69
